# Osteomyelitis and Septic Arthritis of the Upper Extremity in Pediatric Patients

**DOI:** 10.1007/s12178-024-09938-3

**Published:** 2024-12-24

**Authors:** Nnaoma M. Oji, Coleen S. Sabatini

**Affiliations:** 1 Department of Orthopaedic Surgery, Hospital for Special Surgery, New York, NY USA; 2 Department of Orthopaedic Surgery, University of California San Francisco, San Francisco, CA USA; 3 UCSF Benioff Children’s Hospital Oakland, 747 52nd Street, OPC 1st Floor, Oakland, CA 94609 USA

**Keywords:** Pediatrics, Infection, Osteomyelitis, Septic Arthritis, Upper Extremity

## Abstract

**Purpose of Review:**

For pediatric osteomyelitis and septic arthritis, 10–24% of cases occur in the upper extremity (UE). Due to delays in presentation and diagnosis, UE infections are often more complex and severe than infections of the lower extremity (LE). This review evaluates the literature from the past 6 years related to pediatric osteomyelitis and septic arthritis of the UE and provides a guide for professionals managing these conditions in children.

**Recent Findings:**

The shoulder and elbow are the most commonly affected joints, and the humerus is the most commonly affected bone. As with the LE, diagnosis of UE osteoarticular infections is based on clinical evidence, laboratory data, and diagnostic imaging. While *Staphylococcus aureus* is the primary bacteria identified in UE infections, there is an underappreciation of the burden from *Kingella kingae* as a causative organism in culture-negative patients where PCR is not performed. Septic joints should be treated with irrigation and debridement urgently, with subsequent antibiotic therapy for a minimum of 2–4 weeks. For acute osteomyelitis without abscess or concomitant septic joints, antibiotic therapy is standard of care. Methicillin-resistant *Staphylococcus aureus* is associated with more severe infection requiring more surgeries. Various strategies exist for managing segmental bone loss in chronic osteoarticular infections.

**Summary:**

Osteomyelitis and septic arthritis tend to occur less frequently in the UE than the LE but have a devastating impact on the health and quality of life of children around the world. Complete resolution of disease can be achieved through an individualized approach to antibiotic and operative management. Further study is needed to assess the efficacy of aspiration as a primary treatment strategy in UE joints.

## Introduction

Osteomyelitis and septic arthritis are musculoskeletal infections with a variety of manifestations in children. Osteomyelitis is inflammation of bone secondary to microbial infection [1] and septic arthritis is microbial infection of the synovial membrane, joint space, and intraarticular structures [2]. The global burden of osteoarticular infections in children is immense, as annual hospitalization rates continue to increase around the world [3, 4]. Advances in the detection and treatment of both diseases have reduced the prevalence of long-term sequelae in many areas, but chronic disease and permanent disability continue to have devastating impacts on the health and quality of life for children in resource-limited areas across the world [5, 6].

Both osteomyelitis and septic arthritis tend to occur more frequently in the lower extremity (LE) compared to the upper extremity (UE) [7, 8]. Disability in LE infections is often more apparent given that weightbearing and mobility can be impaired. Perhaps due to smaller case numbers and an underappreciation of the disability caused by UE infections, less literature is available for those. As a result, the purpose of this review is to summarize the recent literature related to pediatric upper extremity osteomyelitis and septic arthritis and to provide up-to-date information regarding management of these conditions in children.

## Epidemiology

The overall incidences of pediatric osteomyelitis and septic arthritis have been reported to peak at rates of 80 per 100,000 and 10 per 100,000 respectively [9], but global trends vary significantly depending on geography, patient demographics, and microbial patterns.

### Geography

The literature suggests that UE osteoarticular infections comprise 13–24% of all osteoarticular infections in a diversity of countries [7, 8, 10–15]. A majority of available data reflects single-institution, retrospective case studies, but highlights the importance of understanding these infections when caring for children in any part of the world.

### Patient Factors

With regard to sex, research indicates that osteoarticular infections generally tend to occur more frequently in male infants and toddlers [2, 16]. This aligns with a study of UE septic arthritis, where 66% of the children were male and the median age was 1.7 years [17], and a study of septic arthritis of the shoulder, where 68% of the children were male and 76% were below the age of three [18]. No investigations specifically examine the relationships between socioeconomic factors and rates of UE osteomyelitis and septic arthritis, but widespread studies of osteoarticular infections have found increased rates of hospitalization for American children living in households with a very low median income [7, 8] and, in general, higher rates of osteoarticular infections in low-income countries [19]. The association between low socioeconomic status and higher risk for disease should prompt practitioners to be particularly vigilant evaluating for osteoarticular infections in resource-limited settings.

### Microbial Patterns

Methicillin-sensitive *Staphylococcus aureus* (MSSA) was the most causative organism in a United States (US) study of UE septic arthritis, accounting for 38.8% of positive joint aspirate cultures, though *Streptococcus* was the most commonly identified pathogen in cases of septic shoulder specifically [17]. Both MSSA and methicillin-resistant *S. aureus* (MRSA) were the most frequently cultured organisms in retrospective cohort studies of children with septic elbow arthritis performed in the US and Tunisia [20, 21]. Similar results were reported in studies of septic shoulder arthritis and osteomyelitis of the humerus, but these studies also had culture-negative results ranging from 36.8–66% [18, 22]. Although *S. aureus* continued to be identified as the most prevalent causative agent of osteomyelitis and septic arthritis through 2017 [23, 24], with MRSA becoming ubiquitous globally, the persistence of negative cultures prompted new investigations. Consistent with other recent studies, research from the United Kingdom and Portugal identified *Kingella kingae*, which is often culture-negative, as the most commonly isolated organism in children with septic arthritis [25, 26]. As a result, suspicion for *K. kingae* must be considered, particularly in children younger than three with a relatively mild clinical picture [15, 27].

## Etiology, Risk Factors, Concomitant Infections

The anatomical structure of the pediatric musculoskeletal system makes children more susceptible to disease compared to skeletally mature patients. Seeding of the bone and joint frequently occurs due to hematogenous spread but can also be the result of direct inoculation or contiguous spread [27–30].

The most common sites for hematogenous osteomyelitis seeding are the metaphysis of rapidly growing bones. These areas are highly vascularized and contain leaky vessels with slow blood flow, which enables microbial deposition and proliferation in the capillary loops [31, 32]. In the UE specifically, the relative frequencies of osteomyelitis in the humerus, ulna, and radius are 5–13%, 1–2%, and 1–4% respectively [33]. Hematogenous septic arthritis occurs in a similar fashion, where microbial accumulation in highly vascularized joint synovium leads to infection [27]. Contiguously spread septic arthritis occurs most frequently in the shoulder, elbow, and hip because they have metaphysis that are intracapsular, which allows infections that breach the cortex of metaphyseal bone to enter directly into the joint space [30]. A recent multicenter study of 684 children in the US reported that 10% of septic arthritis cases occurred in the upper extremity, with 53% in the elbow, 41% in shoulder, and 4% in the wrist [17]. In a study that compared septic shoulder and elbow, children with septic shoulder tended to be younger, 1.0 year versus 4.6 years respectively [17].

The relationship between osteomyelitis and septic arthritis has been well described in the pediatric population. Rates of combined infections in the US increased from 0.8 to 1.3 per 100,000 between 1997 and 2012 [3], and a US multicenter study discovered that 46% of children with UE septic arthritis had adjacent musculoskeletal infections and/or bacteremia [17]. While a study of 247 European children from 2003 to 2018 reported that 17% of patients presented with concomitant osteomyelitis and septic arthritis, with 9.5% involving the humerus [25], these rates are lower than those reported in another European study, where concomitant bone or joint involvement was found in 24% of patients with osteomyelitis and 36% of patients with septic arthritis [34]. Higher incidences of concomitant infections are expected in very young children because they have transphyseal vessels that span the growth plate, which facilitate spread [29]. However, research highlighting that rates of concomitant infections among older children were only slightly less than those among younger children suggests that all age groups should be evaluated for concomitant infections [34].

## Diagnosis

Acute osteomyelitis cases often present within 1–3 weeks and are associated with inflammatory bone changes, while chronic infections last more than 4 weeks from the onset of symptoms and are characterized by the presence of necrotic tissue and sequestrum. Septic arthritis often presents earlier in onset due to significant pain in the joint from the onset of the infection [19, 27].

### Clinical Presentation

In patients with long-bone or joint infection, fever, pain, tenderness, and refusal to use/bear weight on the affected extremity are common presenting features. Geographic variation in demographic, clinical, and bacteriological profiles contributes to differences in presentation [35]. For example, pain was the most common symptom in studies of acute osteomyelitis in Nigeria, Taiwan, and Italy, but the percentage of patients with swelling were 62.5%, 87.1%, and 55.4% respectively [10, 36, 37], with some of those differences likely related to the bone involved and amount of soft tissue covering. In those studies, the percentage of cases of UE involvement were 23.5%, 17.1% and 17.4% respectively. Despite this heterogeneity, pseudoparalysis of the involved limb is a relatively consistent feature, appearing in 78–100% of children across numerous studies [20–22, 32, 38, 39]. Therefore, when evaluating a child for an UE infection, pseudoparalysis should be assessed for by assessing active, voluntary motion. Presentation is also variable across age groups, and atypical presentations often observed in young infants, such as refusal to feed, crying, and discomfort with attempted movement, should raise suspicion for an osteoarticular infection [27].

### Laboratory Evaluation

Laboratory assays and bacterial cultures are valuable tools in validating the diagnosis of acute osteoarticular infections. Complete blood count with leukocyte differential; erythrocyte sedimentation rate (ESR); and c-reactive protein (CRP) are the traditional set of laboratory tests ordered in cases of suspected osteoarticular infection, and each of these values is typically elevated in children with acute infection [9]. All of these serological tests were ordered in studies of septic shoulder, septic elbow, septic wrist, and humeral osteomyelitis, demonstrating their role in the diagnosis of osteoarticular infections [17, 18, 20–22, 32, 38, 39]. One specific study of septic shoulder determined that CRP had a 96% sensitivity [38], making it the most reliable predictor of these serological parameters in patients with disease [38]. Surprisingly, blood cultures were only obtained in the UE septic arthritis study [17], the study of humeral osteomyelitis [22], and one study of septic shoulder [18]. On the other hand, culture of aspirated synovial fluid was included in all studies of septic elbow [20, 21], septic wrist [39], UE septic arthritis [17], and one study of septic shoulder [32], which highlights the importance of arthrocentesis in the diagnostic process for septic arthritis.

### Microbiology Analysis

Culture results were negative in more than 60% of patients in studies of UE septic arthritis [17], humeral osteomyelitis [22], and septic shoulder [38], illustrating the limitations of microbial cultures in identifying causative organisms. These high frequencies have led to the development of new approaches for identifying pathogens responsible for acute osteoarticular infections [22, 38]. Studies have shown that polymerase chain reaction (PCR) has the highest pathogen detection rates in children with septic arthritis [40], which has enabled this modality to play a critical role in identifying *K. kingae* as a common cause of pediatric septic arthritis [25, 26]. Furthermore, PCR has been found to result in shorter treatment durations [34]. Two additional tools discussed in the literature include serum procalcitonin (PCT) and gram stain. A meta-analysis comparing serum PCT and CRP found that PCT was more valuable in distinguishing septic arthritis from non-septic arthritis, but the applicability of these results to children is limited, as only one of the ten studies included pediatric patients [41]. In contrast, a recent systematic review suggested that serum PCT may serve as a biomarker for osteomyelitis but not septic arthritis, though additional studies are needed to verify its diagnostic parameters [42]. Gram stain has proven to be a poor clinical screening tool for the detection of septic arthritis, as shown in a study of 302 children where ${~}^{1}\!\left/ \!{~}_{5}\right.$ of patients were misdiagnosed by Gram stain [43].

### Diagnostic Imaging

Imaging can further distinguish the presence of osteoarticular infections as well as the extent of spread.

#### X-Rays

**Fig. 1.**
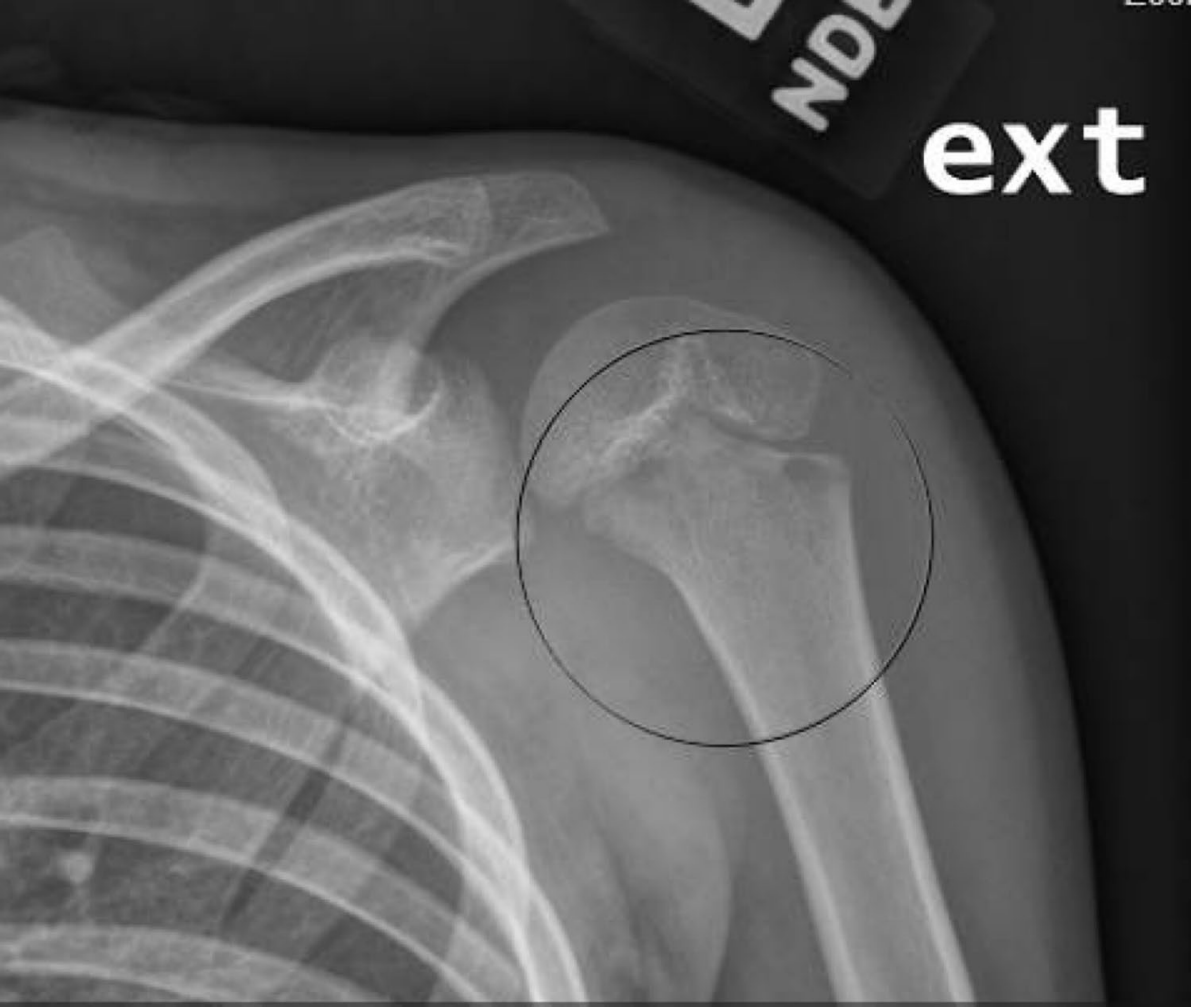
8-year-old male with > 1 week of worsending left shoulder pain and limited range of motion after recent sinus infection. Note the ill-defined lucencies in the metaphysis

**Fig. 2:**
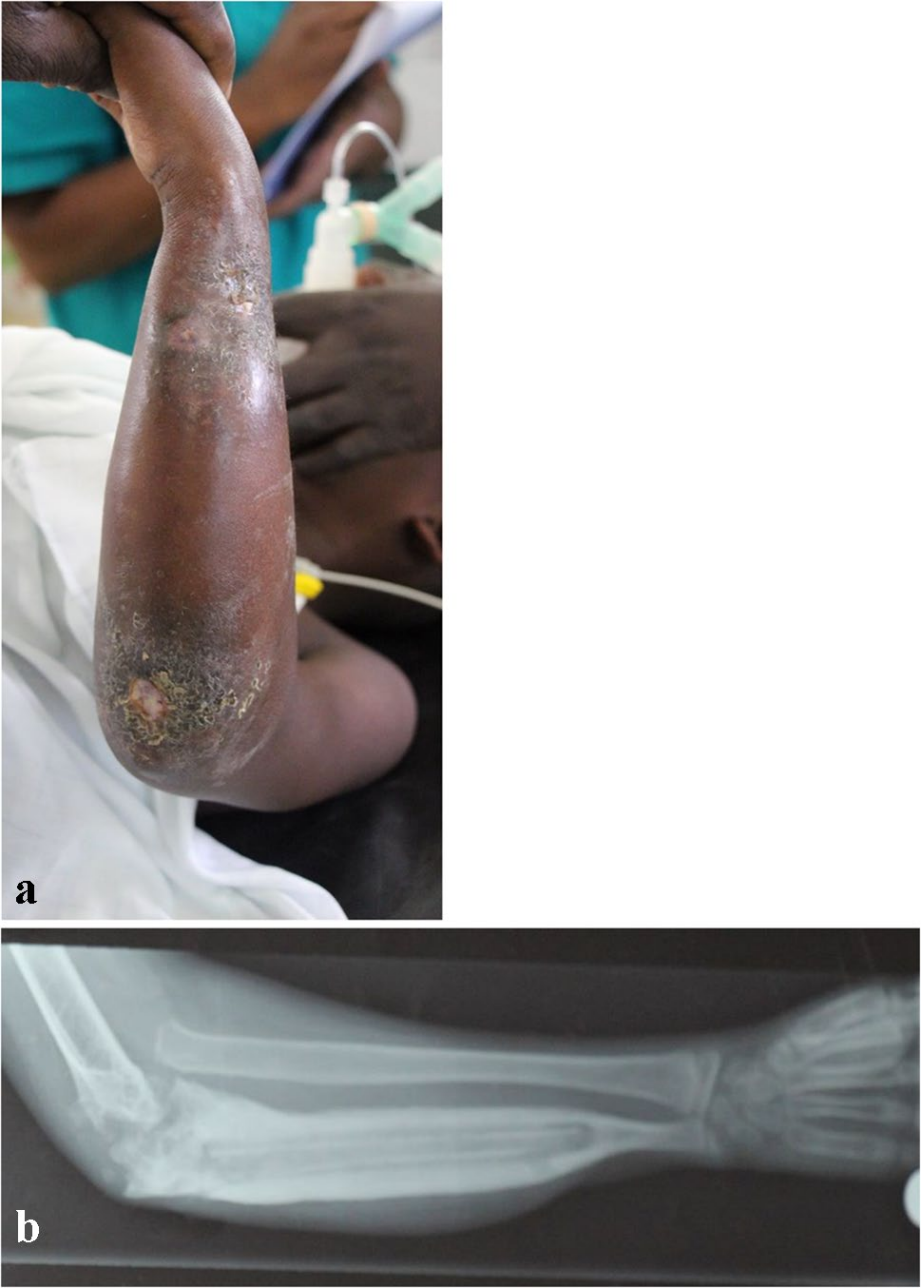
**a** clinical photo of a child with two areas of draining sinus due to underlying chronic osteomyelitis. **b** AP xray of the forearm demonstrating a large sequestrum in the ulna with surrounding involucrum. The radiocapitellar joint is dislocated and the ulnohumeral joint shows degenerative changes, consistent with an untreated septic arthritis

Conventional radiographs are often the first form of imaging obtained in the evaluation of both osteomyelitis and septic arthritis [30, 33]. Relevant findings are listed in Table 1, though bony changes often take 7–14 days to develop in osteomyelitis (See Fig. 1) While only 27% of patients had a positive radiographic finding in a study of humeral osteomyelitis [22], 67–100% of children in studies of septic elbow were found to have effusion on radiographic imaging [20, 21]. These results suggest that radiographs may not be the most sensitive imaging technique for the entire UE, yet their low cost, rapid results, and ability to rule out other conditions support their continued use [30, 33]. In chronic osteomyelitis, x-rays are helpful to define the sequestrum and assess the involucrum; lucency within the canal is suggestive of intraosseous abscess (Fig. 2a and b).

**Table 1:** Diagnostic Imaging in Septic Arthritis and Osteomyelitis

	Indications	Relevant Information/Findings	Limitations
X-Ray	Primary screening tool for osteoarticular infections	Tissue swelling, joint space widening, osteolytic findings, irregularity of bone profile (e.g. shape, structure, etc.), periosteal reaction [44]. In chronic osteomyelitis, helpful to visualize sequestrum and assess the development of involucrum	Lower sensitivity; unable to detect findings early in disease process (first 2 weeks). Not specific
Bone Scintigraphy	Detection of osteomyelitis when symptoms are unable to be localized	Increased tracer uptake in all three phases	Non-specific findings; exposure to radiation
Computed Tomography (CT)	Not commonly used in children when MRI is available	Can show even subtle changes in the bone, superior bony resolution to MRI – shows periosteal reaction, cortical destruction, and sequestrum	Not modality of choice in children due to radiation exposure. Does not show marrow edema, so not helpful for early infection
MRI	Presumed osteoarticular infection when symptom onset in < 2 weeks and trauma can be excluded, assessment of contiguous infection for surgical planning	Active inflammation (low signal on T1-weighted and a high signal on T2-weighted) [5]; effusion, abscess, pyomyositis, subperiosteal abscess, cortical irregularity [18, 38]. Fluid-sensitive sequences helpful for detecting infection and inflammation. Gadolinium used to define possible abscesses and sinus tracts	May require sedation/general anesthesia; high cost; results often not available immediately
Ultrasound	First-line screening tool for osteoarticular infections, particularly septic arthritis	Tissue swelling, joint effusion, and synovial thickening across all age groups; increased vascularization in children > 5 years of age [44]	Unable to detect findings < 24 h of symptom onset; user dependent; unable to distinguish between sterile and infective fluid accumulations

#### Bone scintigraphy

In children unable to verbalize the location of their symptoms, bone scintigraphy of the entire body is another approach for determining the presence of osteomyelitis specifically [45]. 70% of tested children in a study of humeral osteomyelitis had positive results on bone scintigraphy [22], but this technique exposes children to larger amounts of radiation and was not utilized in any other recent studies of UE osteoarticular infections.

#### Ultrasound

Ultrasound is also used to evaluate the presence of osteoarticular infections in children. Since this technology is non-invasive, portable, inexpensive, and provides real-time results, it can serve as first-line imaging alongside radiographs [46]. Relevant findings, which often represent changes to the joint rather than the bone itself, are listed in Table 1. In a study of septic shoulder, 71% of cases had positive ultrasound findings, with 67% having signs of septic arthritis with no bone involvement and 20% having signs of arthritis with concurrent osteomyelitis [38]. The hallmark of septic arthritis on ultrasound is the presence of joint effusion, which provides immediate opportunities for aspiration, but does not necessarily allow it to be distinguished from inflammatory conditions of the joint [5] Similar to radiographs, ultrasound may report negative findings within 24-h of symptom onset, as changes to joint or bone may not yet be detectable so early in the disease [5].

#### Magnetic Resonance Imaging (MRI)

**Fig. 3:**
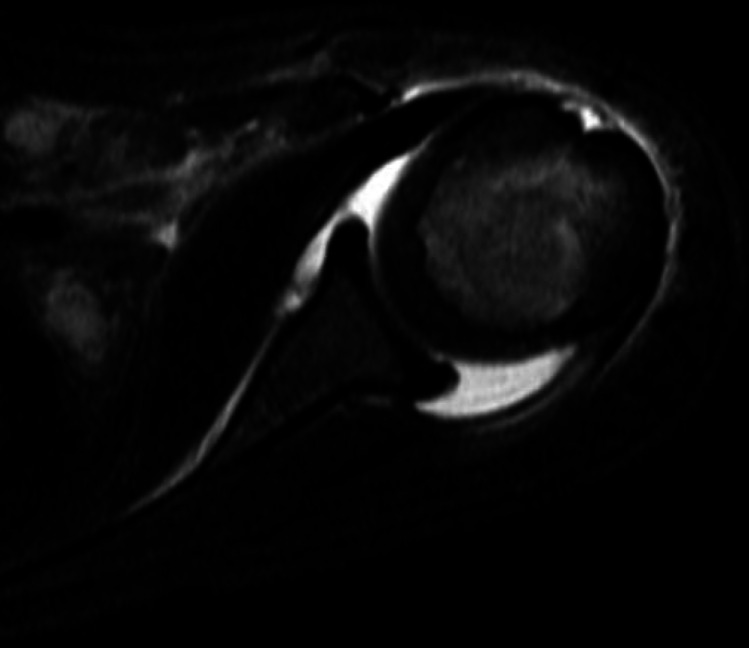
MRI of a shoulder showing joint effusion in setting of a septic joint

MRI provides excellent visualization of both soft tissue and cortical structures and has been found to be the most sensitive and specific imaging modality for osteoarticular infections [45]. In one humeral osteomyelitis study and two septic shoulder studies, positive MRI findings were recorded in 85%, 100%, and 100% of tested patients respectively [18, 22, 38]. An investigation of imaging modalities recommended that MRI be considered in cases of presumed infection when the onset of symptoms is less than two weeks (as changes will not likely be seen on x-ray) and trauma can be excluded [34]. Relevant MRI findings are listed in Table 1, including edema. (See Fig. 3). MRI also avoids radiation exposure and helps guide surgical treatment by assessing the extent of infection. The length of time to obtain results and potential need for sedation pose challenges [30]. Further, the lack of access to this expensive tool in resource-limited areas limits the scope of its benefits.

##### Aspiration

Arthrocentesis can provide both diagnostic and therapeutic utility in the management of septic arthritis, with 72% of aspirate cultures having positive results in a study of UE septic arthritis. Aspiration can be performed rather quickly and moderate sedation is the most common form of anesthesia used in UE aspiration [47]. Anterior and dorsal approaches have been utilized in studies of septic shoulder [18] and wrist [39] respectively. A lateral approach was employed in two studies of septic elbow [20, 21], but posterior aspiration into the olecranon fossa is quite simple and does not put nerve or articular cartilage at risk. 88% of UE joint aspirations are completed based on anatomic landmarks rather than image guidance [47]. Since aspiration is not a feasible non-surgical approach for collecting tissue samples in cases of osteomyelitis, some institutions have employed image-guided biopsy techniques, which has demonstrated increased pathogen identification and reduced time to definitive antibiotic selection [48, 49].

## Treatment of Acute Infection

The goal of treating acute osteoarticular infections in children is to provide prompt therapeutic interventions that stabilize the patient, prevent disseminated disease, and avoid devastating long-term complications. This task is best undertaken with a multidisciplinary team composed of pediatricians, infectious disease specialists, musculoskeletal radiologists, pediatric orthopaedic surgeons, and ancillary staff [50]. Antibiotics serve as the foundation for the non-operative treatment of acute osteoarticular infections, and the most effective treatment regimens are based upon patient age, extent of disease, local sensitivities, and directed therapy with pathogen identification [46]. Historically, antibiotic therapy was initiated after culture samples were taken in an effort to improve cultural yields, but two retrospective studies questioned this, showing that tissue culture sensitivities of musculoskeletal infections were not significantly decreased by antibiotic administration prior to the collection of cultures [51, 52]. While this finding suggests that empiric therapy with broad spectrum coverage can be started immediately at the time of diagnosis, randomized-controlled trials are needed to confirm this approach.

The choice of empiric therapy continues to be an area of debate. For example, in separate studies of septic shoulder, the empiric antibiotics of choice were clindamycin in one study [18] and vancomycin in the other [32]. While both of these agents are effective against MRSA infections [53], an individualized approach that considers local guidelines, microbial resistance patterns, and patient factors should be employed when initiating empiric antibiotic therapy [31, 54]. Additionally, antibiotic therapy should be narrowed as soon as sensitivity data is available to limit unintended consequences of antimicrobial use [55]. Commonly used antibiotics in studies of UE infections include amoxicillin clavulanate [22], cefuroxime [38], and oxacillin–gentamycin [21]. A third-generation cephalosporin should also be considered alongside empiric antistaphylococcal therapy given the increased prevalence of *K. Kingae* in toddlers [15, 29].

In uncomplicated cases of osteoarticular infections, 3–4 days of intravenous antibiotics are recommended prior to transitioning to oral therapy, though this decision is contingent upon clinical improvement, reduction in inflammatory markers, and the ability for the appropriate antibiotic to be taken orally [31, 54]. 2–4 weeks of oral therapy is often appropriate if inflammatory markers normalize and clinical resolution is evident; otherwise, the course of antibiotics should be extended to 6 weeks total [31]. This aligns with studies of humeral osteomyelitis and septic elbow, where average durations of oral therapy were approximately 2.6 weeks and 4.4 weeks respectively [20, 22]. Alongside antibiotics, recent investigations have suggested the use of corticosteroids as an adjunctive therapy in the treatment of septic arthritis. A meta-analysis reported that corticosteroids shortened hospital stays, the total duration of antibiotic therapy, and days to CRP normalization [56], but a separate Cochrane review determined the evidence for corticosteroids in children with septic arthritis to be of low quality [57].

Operative treatment of osteoarticular infections includes drainage, decompression, irrigation, and debridement [30]. In addition to aiding in source control, enabling antibiotic penetration at the site of infection, and cleaning the surrounding tissue, surgery enables intraoperative collection of samples that can be used to identify caustic organisms and guide targeted antibiotic therapy [54]. This has been found to prevent disease progression; morbidity, such as osteonecrosis and cartilage damage; and mortality [16, 30, 54].

Septic arthritis has the potential to rapidly evolve into systemic disease, so urgent removal of inflammatory products is necessary [45], especially if pus is present [2]. This can be done with aspiration, arthroscopy, or arthrotomy [45]. Arthrotomy resulted in positive short-term outcomes and low complication rates in a study of UE septic arthritis [17], and this approach was also the most commonly used intervention in studies of septic shoulder [18, 32, 38], elbow [20, 21], and wrist [39]. However, arthroscopic lavage was shown to be equally effective in treating septic shoulder [38, 58]. Furthermore, a systematic review comparing arthrotomy and aspiration for septic shoulder and elbow determined that both approaches, followed by intravenous antibiotics, were capable of achieving good clinical results, though aspiration was over three times more likely to result in additional procedures [59].

For acute osteomyelitis, surgical drainage is often necessary to resolve infections that are not responsive to antibiotic therapy for 48–72 h or that develop complications (e.g. moderately large abscesses, concomitant septic arthritis, etc.) [16]. In a study of humeral osteomyelitis, surgery was indicated for 53% of the patient’s because they had radiological signs of subperiosteal collection or potential adjacent septic arthritis [22]. Surgical procedures depended on intraoperative findings but often consisted of subperiosteal abscess irrigation and drainage [22]. It is important to note that variations in surgical management of acute osteomyelitis are often institution driven, and that incorporation of various clinical factors in decision-making can affect rates of surgical intervention [60].

Additional procedures are warranted if the following situations occur subsequent to surgical intervention and antibiotic therapy: progression of clinical symptoms, continued drainage, or persistence of elevated CRP levels [16, 27, 30]. In a study of septic shoulder, where arthrotomy was exclusively used, MRSA-infected patients were significantly more likely to require additional operations to eliminate infection than those with non-MRSA infections [18]. Furthermore, a study of subperiosteal abscesses, including 11 cases in the UE, found that the combination of intramedullary decompression/debridement and abscess drainage decreased the risk for repeat surgical intervention when compared to abscess drainage alone [61].

## Outcomes

Prompt diagnosis and treatment of osteoarticular infections are essential to achieving good outcomes and preventing complications [9, 29, 30]. Although treatment was initiated on the day of admission in 90% of children with humeral osteomyelitis, two patients developed multiorgan failure secondary to sepsis and a third developed a fixed flexion deformity secondary to avascular necrosis of the trochlea [22]. These three children, in addition to four others, had concomitant septic arthritis, which was associated with significant delay to presentation and a more severe form of disease [22]. In a study of UE septic arthritis, avascular necrosis, recurrence of infection, repeat irrigation and debridement, and a pathologic fracture all occurred in patients with concomitant osteomyelitis [17]. Another study of septic shoulder found high rates of concomitant disease, with 75% of patients having adjacent osteomyelitis on MRI [18]. They did not find an association between concomitant disease and poor outcomes, but they did discover that MRSA-infection, compared to other organisms, was a predictor of more severe disease course, negative outcomes, and adverse sequelae [18], which aligns with other literature [2, 6, 29]. High rates of concurrent osteomyelitis were observed in a study of septic elbow, where 58% of septic arthritis patients had osteomyelitis [21]. Delayed diagnosis in two of these patients resulted in readmission and repeat surgery to treat persistent symptoms [21], and a third patient became septic and died from multiorgan failure during their admission [21]. While no patients became septic in a separate study of septic elbow, 20% of patients were found to have elbow stiffness at their last follow-up [20].

In general, adverse outcomes are related to severe disease, delayed therapy (> 4–5 days from onset of symptoms), neonatal infections, and location of infection [9, 27]. Multiple studies of pediatric osteomyelitis have shown that complicated cases occur more frequently in the UE when compared to the LE [10, 62], which is likely due to children exhibiting a limp with LE infection and therefore receiving medical attention earlier in disease course [62]. Despite this, research into pediatric septic arthritis suggests that the hip has the worst outcome of all joints [2], especially in neonates [63]. In an effort to standardize care delivery and improve pediatric outcomes, classification systems, clinical care guidelines, and prediction tools that proactively identify osteoarticular complications and assess disease severity have been developed [62, 64–67]. For example, one study showed that sepsis and hypergammaglobulinemia were associated with higher frequencies of late sequelae (e.g. reduced range of motion, permeant deformity, etc.) [68] while another discovered that failure to reduce CRP by 50% at hospital day 4 or 5 predicted both acute and chronic complications [69]. A prediction algorithm utilizing age, CRP, duration of symptoms, platelet count, and absolute neutrophil count (ANC) was capable of distinguishing septic arthritis with adjacent infection, such as osteomyelitis, from isolated septic arthritis [70]. The authors suggested that children meeting ≥ 3 of these criteria may benefit from preoperative MRI, which aligns with studies of septic shoulder and elbow that recommend preoperative MRI to detect concomitant osteomyelitis and assist in surgical decision making [18, 21].

## Chronic Infection

Access to appropriate medical care and follow-up is often enough to prevent acute osteoarticular infections from progressing to chronic disease. While one study showed that a majority of children with acute osteomyelitis did not require follow-up beyond the initial treatment period [6], other studies recommend follow-up within 2–6 weeks of discharge to assess clinical improvement [27, 29], with prolonged monitoring reserved for patients with complications or infections near the growth plate [1, 30]. The prevention of long-term sequelae associated with chronic disease is paramount in avoiding permanent disability. Septic arthritis is often a surgical emergency given destruction of cartilage and its ability to rapidly evolve into systemic disease [21]. As a result of its acute disease course, evidence regarding the chronicity of this condition is limited when compared to osteomyelitis.

**Fig. 4:**
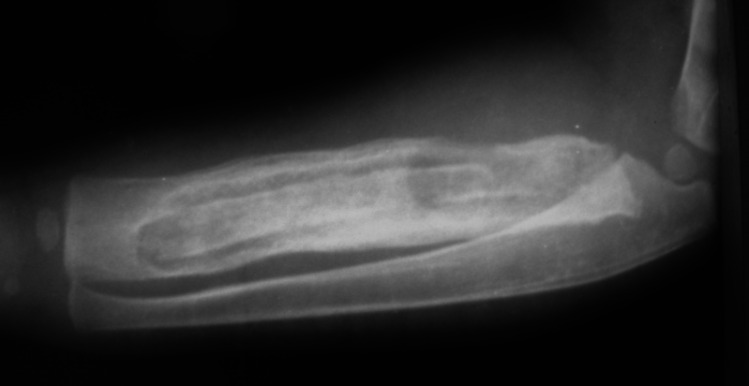
An approximately 2-year-old child with chronic osteomyelitis of the radius. Note the sequestrum in the intramedullary canal with the surrounding involucrum

**Fig. 5:**
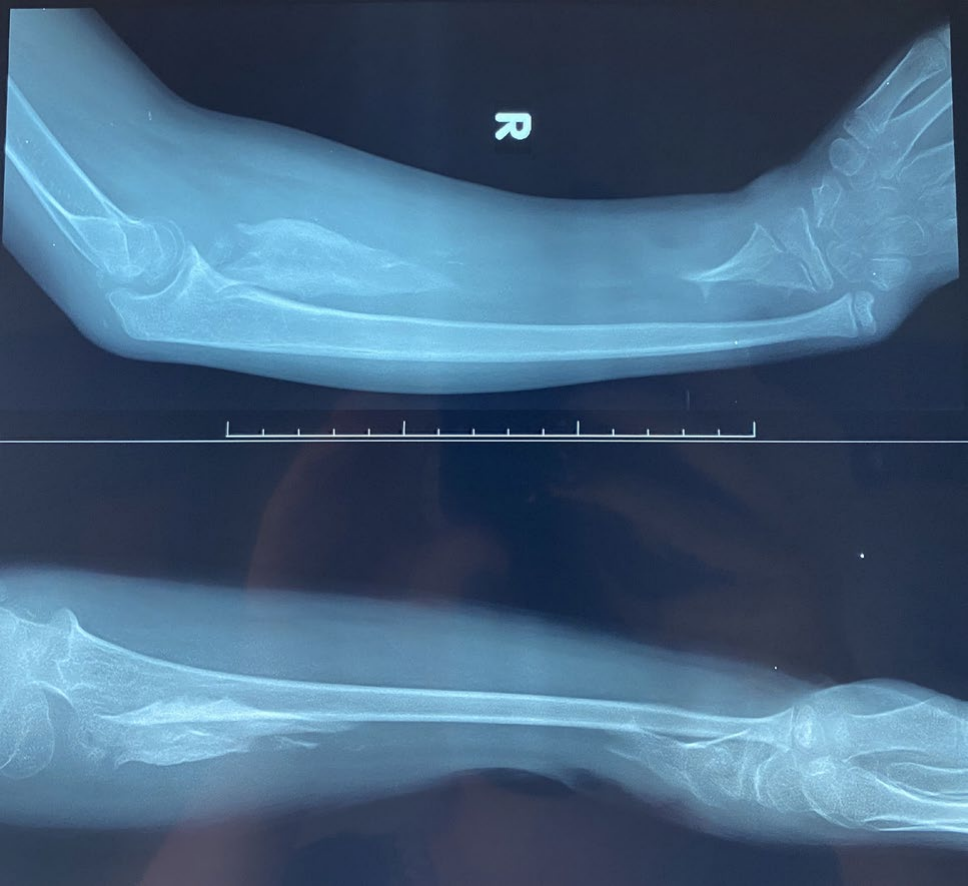
Child with delayed presentation chronic osteomyelitis with large segmental bone defect from either extruded or surgically excised sequestrum of the diaphysis, without involucrum

Persistent symptoms and the development of necrotic bone are hallmarks of chronic osteomyelitis [28]. The necrotic bone, referred to as the sequestrum, is surrounded by pus and reactive bone sclerosis. There is usually new bone forming as well, the involucrum. (See Fig. 4) Necrosis may appear 1–2 weeks prior to the development of these features [28, 33]. Rates of chronic osteomyelitis tend to be higher in resource-limited areas due to delays in access to care where acute osteomyelitis goes undertreated or without treatment [71]. An epidemiological study of osteomyelitis in Nigeria found 65.8% of the children had chronic disease, with 84% of those children having an initial onset of symptoms that were either neglected or mistreated [36]. In contrast, a study of chronic osteomyelitis in New Guinea determined that 73% of the cases were caused by inadequate treatment of open fractures [71]. 17% of the infections in this study were of the forearm, 10% were of the humerus, and 83% of the *S. aureus* strains were MRSA [71]. The authors of this study were able to achieve successful treatment in 95% of cases using radical debridement and antibiotics [71]. In a separate study in Rwanda, locally-made, biodegradable, calcium-sulfate bone cement pellets impregnated with antibiotics were successful in treating 95% of patients with chronic osteomyelitis, including 30 children [72], but there was not a comparison group of those treated without antibiotic impregnated calcium sulfate. Of note, no recent studies show improved outcomes in children with use of antibiotic beads/cement relative to other treatments. In cases of post-osteomyelitic forearm segmental defects (see Fig. 5), fibular strut with additional bone grafting has been shown to be an effective reconstructive method [73, 74], as it enables union and restores a majority of forearm function. Vascularized fibula flaps have also been shown to be a valid approach for treating UE post-osteomyelitic bone defects [75]. Bone transport and other segment defect filling techniques have also been employed, but there is not recent literature available to review. Further research into the factors contributing to the persistence of chronic infections in resource-limited areas is essential to developing prevention and treatment strategies that improve outcomes in patients and improve musculoskeletal outcomes for future generations.

## Conclusion

Osteomyelitis and septic arthritis tend to occur less frequently in the UE than the LE, but these infections often present later and can be more complex, can result in permanent disability, and have a devastating impact on the health and quality of life of children around the world. The anatomical structure of the pediatric musculoskeletal system makes children more susceptible to disease when compared to older populations, while being less able to articulate their symptoms. Practitioners must have infection on their differential when evaluating children and be particularly vigilant in considering osteoarticular infections, including MRSA and *K. Kingae*, in resource-limited settings in order to proactively prevent progression to chronic disease. Pseudoparalysis, elevated inflammatory markers, microbiological analysis, and characteristic imaging findings confirm the diagnosis of osteomyelitis and septic arthritis, and complete resolution of disease can be achieved through an individualized approach to antibiotic and operative management. This review critically evaluates the literature from the past several years related to pediatric osteomyelitis and septic arthritis of the UE and provides a guide for professionals managing these conditions in children.

## Data Availability

No datasets were generated or analysed during the current study.
